# Biomodifying the ‘natural’: from Adaptive Regulation to Adaptive Societal Governance

**DOI:** 10.1093/jlb/lsac018

**Published:** 2022-06-30

**Authors:** Miranda Mourby, Jessica Bell, Michael Morrison, Alex Faulkner, Phoebe Li, Edison Bicudo, Andrew Webster, Jane Kaye

**Affiliations:** Centre for Health, Law and Emerging Technologies (HeLEX), Faculty of Law, University of Oxford, Oxford, UK; School of Law, University of Warwick, Coventry, UK; Centre for Health, Law and Emerging Technologies (HeLEX), Faculty of Law, University of Oxford, Oxford, UK; School of Global Studies, University of Sussex, Brighton, UK; School of Law, Politics & Sociology, University of Sussex, Brighton, UK; Department of Science, Technology, Engineering and Public Policy (STEaPP), Faculty of Engineering Science, University College London, London, UK; Science and Technology Studies Unit (SATSU), Department of Sociology, University of York, York, UK; Centre for Health, Law and Emerging Technologies (HeLEX), Faculty of Law, University of Oxford, Oxford, UK

**Keywords:** synthetic biology, regenerative medicine, Adaptive Governance

## Abstract

Biomodifying technologies—such as gene editing, induced pluripotent stem cells, and bioprinting—are being developed for a wide range of applications, from pest control to lab-grown meat. In medicine, regulators have responded to the challenge of evaluating modified ‘natural’ material as a therapeutic ‘product’ by introducing more flexible assessment schemes. Attempts have also been made to engage stakeholders across the globe on the acceptable parameters for these technologies, particularly in the case of gene editing. Regulatory flexibility and stakeholder engagement are important, but a broader perspective is also needed to respond to the potential disruption of biomodification. Our case-study technologies problematize basic ideas—such as ‘nature’, ‘product’, and ‘donation’—that underpin the legal categories used to regulate biotechnology. Where such foundational concepts are rendered uncertain, a socially responsive and sustainable solution would involve exploring evolutions in these concepts across different societies. We suggest that the global observatory model is a good starting point for this ‘Adaptive Societal Governance’ approach, in which a self-organizing network of scholars and interested parties could carry out the multi-modal (meta)analyses needed to understand societal constructions of ideas inherent to our understanding of ‘life’.

## I. INTRODUCTION

### I.A. Background

Biotechnology has long challenged our understanding of the boundary between ‘nature’ and ‘artifice’. In recent years, however, a new generation of techniques—that we call ‘biomodifying technologies’—has made life at the biological level of cells, genes and proteins malleable, and amenable to more fundamental reformulation.^1^ For example, gene editing through mechanisms such as CRISPR can be seen as going beyond mere replacement of genetic material with other ‘naturally occurring’, ‘donated’ material, and instead constituting alterations, which (when used in people) have implications for fundamental human identity.^2^

Induced pluripotent stem cell (‘iPSC’) technology—which enables ordinary cells of the adult body to be ‘reprogrammed’ into any other kind of cell—and the ‘bio-printing’ of human tissue similarly present new challenges to how we consider the ‘donor’ of an iPSC, and whether printed human tissue should be treated as a ‘product’. These concepts are key not only to how we understand biotechnology, but also how we regulate its use. Laws governing the use of biomodifying technologies serve as a delicate bridge between social understandings of ‘biology’ and ‘modification’, and the reality of how these techniques work. Where societal concepts and regulated activity no longer align, this threatens the clarity and legitimacy of the law governing biomodifying technology.^3^

Societal framings of ‘nature’, ‘products’ and ‘donors’ may be tentative and heterogeneous, and will find expression outside academic, policy or regulatory discourse—for example in popular culture and on social media. Tracing these developments with sufficient accuracy to provide a reliable barometer for the law is thus a complex proposition. Without this work, however, attempts to gauge the alignment between the regulation of these emerging technologies, and public ideas of their constituent concepts, involve a significant amount of guesswork. Attempts have been made at public engagement on the regulation of biomodifying technologies, and we will argue that this forms part of a more evidentially flexible and socially responsive approach to licensing we will refer to as ‘Adaptive Regulation’. However, we suggest these attempts (while valuable) do not get to the heart of how people in different social contexts understand the concepts, which form the basic building-blocks of laws governing biotechnology.

We believe that the answer lays instead in what has been termed (particularly in an ecosystem-management context) ‘Adaptive Governance’: governance informed by a commitment to social learning through self-organizing networks. However, it is important that such networks expose perspectives across broad sections of society, and on fundamental conceptual questions. Jasanoff and Hurlbut have called for a (now established) observatory of international scholars to track conceptual developments around gene editing.^4^ We endorse this measure, but argue that a broader scope of conceptual monitoring is required. Jasanoff’s scholarly network was proposed, primarily, to discuss the limits and direction of research into gene editing.^5^ Here, we are concerned with how people’s understanding of foundational concepts aligns with their framing in national laws. This would help to (re)assess key explicit or implicit definitions within the law as it is developed to regulate biomodifying technologies.

Clearly, the social, moral and conceptual challenges posed by biomodifying technologies cannot be resolved in a series of disconnected, one-off engagement exercises.^6^ The latter is often all a public body is able to conduct in its attempts to bring its work in line with social attitudes. An observatory could usefully explore how different publics understand the concepts that underpin our attempts to regulate biomodifying technologies, and thus the acceptable boundaries between ‘natural’ and ‘artificial’ in medicine. This would help take us from Adaptive Regulation (ie a flexible approach taken by regulators^7^) to Adaptive ‘Societal’ Governance (‘ASG’), in which self-organizing networks investigate public imaginings of the ‘bio’ and the ‘modified’ through techniques such as studies of social media, ethnography and discourse analysis. The proposition of ASG as a response to biomodifying technologies is the main contribution of this paper.

The next section outlines the idea of ASG, and explains generally why it is needed for biomodification. Section III considers EU regulation of medicinal biomodification to show the strengths and limits of Adaptive Regulation in this context. Section IV then demonstrates how our particular case-study technologies would benefit from an ASG approach. The authors will be able to offer qualitative data to this end in future papers, but here seek to demonstrate the scale of the challenge, and why an international ASG approach is needed as a benchmark for national laws governing biomodifying technology.

### I.B. Methods and Scope

The term ‘biomodifying technologies’ has been taken from the title of two large interdisciplinary projects, funded by the Economic and Social Research Council and the Leverhulme Trust in 2017–2021. They have been conducted in tandem by researchers at the universities of Oxford, Sussex and York, involving a mixture of empirical and socio-legal theoretical research.

The concept of ‘biomodifying technologies’ was developed to capture a particular type of technique, developed through life sciences research, which enables scientists to modify living biological tissue in a directed fashion. The three case-study technologies of the Leverhulme Trust funded ‘Governing Biomodification’ project—gene editing, 3D bioprinting, and induced pluripotent stem cells—are only the most recent examples of this type of technology.

Much of twentieth century biology, and the biotechnology industry, is based on older biomodifying technologies such as cell culture, recombinant DNA, in vitro fertilization (IVF), cloning (somatic cell nuclear transfer) and the polymerase chain reaction (PCR).^8^ While these technologies all derive from different times, places, and avenues of research, they are united in that they all, in different ways, make ‘life’ amenable to being broken down and reformed in new ways through human intervention.^9^ They can be summarized as follows:

1. *iPSC technology*, which enables ordinary cells of the adult body to be ‘reprogrammed’ so that, like the cells of an early embryo, they can become any type of cell in the body, eye, liver, heart and so on.
2. *Gene editing* describes the alteration of DNA using molecular tools.^10^ This can be applied to human beings in vivo for therapeutic purposes.
3. *Bioprinting* takes the concept of 3D printing and applies it to living organic biomaterials. Instead of creating objects from multiple layers of plastic or metal, bioprinting requires a specialized printer, a ‘bioink’ with living cells embedded in a supporting fluid; this can include human cells for transplantation.

Biomodifying technologies are now deployed in a range of sectors. Gene editing can be used to produce genetically modified plants and animals, as well as implementing ‘gene drive’ mechanisms as a tool to control the populations of undesirable ‘pest’ organisms and invasive species,^11^ while stem cell technology and bioprinting are both implicated in the endeavor to create so-called ‘lab-grown’ or ‘in vitro’ meats as an alternative to traditional livestock farming.^12^ Our focus, however, is on their use in novel therapies. The challenges presented by the use of biomodification in medicine are not necessarily unique to this sector, but questions of identity and nature are particularly complex and acute when it is human beings that are ‘modified’. In-human therapeutic uses thus provide important case studies for the importance of societal perspectives for the sustainable translation of these technologies.

A full review of all national laws governing biotechnology—even just in a medical context—would be beyond the scope of this paper. We focus primarily on European Union legislation as the EU has, in the Advanced Therapy Medicinal Product Regulation, made attempts to regulate some biomodifying technologies as in-human therapies. It is thus a useful example of how, even with significant legislative provision, regulatory innovation alone cannot address the social dimensions of the challenge to foundational concepts. But the need for ASG to provide benchmarks against which to measure the law is pertinent beyond the EU context, as biomodifying technologies are in development and use worldwide.

The EU regulatory frameworks are not presented in their entirety. This is partly because they have been comprehensively overviewed elsewhere.^13^ Also, our present argument depends not so much on a complete picture of the relevant laws, as on an illustration of their uncertain interaction with biomodifying technologies. As such, we have identified elements of the regulatory framework we consider to be of greatest relevance to demonstrate the distinction between Adaptive Regulation and Adaptive ‘Societal’ Governance. This is explored in the next section.

## II. ADAPTIVE REGULATION VS ADAPTIVE SOCIETAL GOVERNANCE

### II.A. Regulation and Governance

The three central ideas we discuss in this paper—Adaptive Regulation, Adaptive Governance, and Adaptive Societal Governance—are all responses of different actors to different kinds of the uncertainty created by novel technologies. The difference between them can be summarized as follows:

**Adaptive Regulation**: attempts by regulators to be more flexible when assessing novel technologies in response to *scientific & evidential uncertainty.* This flexibility may be supported by allowances made in the relevant law.

**Adaptive Governance**: social learning through self-organizing networks (ie a network not limited to agents of the state) to manage a common resource disturbed by *uncertainty in the environment*. The ‘environment’ in question could be physical, or a socio-technical ‘eco-system’.

**Adaptive Societal Governance** (‘ASG’): an emphasis within this paper—the idea that social or scholarly learning within a self-organizing network should be open to wider society, by prioritizing multi-disciplinary work that teases out public understandings of key concepts. This is a response to *uncertainty in foundational concepts.*

This section explains how we have arrived at these definitions. This is not a straightforward process, as the terms ‘regulation’ and ‘governance’ are notoriously difficult to define, let alone delineate. In her highly influential ‘Critical Reflections’, Julia Black defines regulation as:

*the sustained and focused attempt to alter the behavior of others according to defined standards or purposes with the intention of producing a broadly identified outcome or outcomes, which may involve mechanisms of standard-setting, information gathering and behavior modification.* ^14^

The ‘cardinal factor’ of Black’s definition of regulation is the *intention* to regulate—to steer the behavior of others.^15^ ‘Governance’, by contrast, can be understood as broader than government, and also encompassing the creation of rule and order through social practices (for example, by a network).^16^ This distinction is crucial for our delineation between Adaptive Regulation and Adaptive (Societal) Governance: whether the intention is to change behavior of technology producers and users (‘Regulation’), or to create a framework for new social order through mutual learning (‘Governance’). The ‘Adaptive’ element that links all three concepts is an intentional adjustment in response to socio-technical uncertainty.

We therefore use the term ‘Adaptive Regulation’ to cover a whole host of innovative practices on the part of regulators around the world, adopted with the aim of evaluating novel technologies in a way that manages the heightened challenge of amassing adequate evidence of safety and efficacy. Adaptive Regulation in EU medicinal product evaluation is discussed in Section III. While we are broadly supportive of Adaptive Regulation, we argue in Section IV that our case studies require adaptive practices to be situated within ASG’s broader framework of reflection on the concepts that underpin and give meaning to the relevant laws and regulations.

An argument for ASG thus goes beyond the suggestion that regulatory agencies should engage with a wider cast of stakeholders in their adaptive practices. As Black herself observes:

*Much discussion of decentered techniques of regulation is still state-orientated... Thus how actors other than the state might be harnessed in the design of hybrid or ‘post-regulatory’ mechanisms usually is debated in the context of how the state can best act to further public policy objectives.*

In other words, calls for public bodies to consult with more stakeholders through formal engagement exercises do not move the mode of governance away from a state-orientated model. The above references to the ‘state’ are broad in nature, given the level of abstraction on which Black’s arguments are made. For our present purposes, we would include as ‘state-orientated’ the activity of all of those—including transnational bodies^17^ and private companies^18^—acting on functions set out in statute or legislation, as these are still important centers of formal power, even if they are not directly (or exclusively) linked to a single nation state. As long as these centers of formal power are the main locus and driver of activity, calls for a more inclusive conversation around advanced therapies still fall within the remit of Adaptive Regulation.

When we come to review the adaptive licensing trailed by the European Medicines Agency (‘EMA’) in Section III, we will see certain points arising in much of the surrounding commentary that echo the above quotation:

1. Advanced therapies are of benefit to patients, as long as they can be translated safely and effectively (the ‘broadly defined outcome’);
2. Data to support claims of safety and efficacy can be more difficult to obtain for these types of treatments (the ‘regulatory challenge’);
3. More flexible, or adaptive, evidential standards can therefore be used to help translate these technologies into therapies (‘mechanisms of standard setting’);
4. In designing these adaptive standards, regulators should engage with ‘key stakeholders’ such as payers (eg commissioners or insurers, depending on the national healthcare system),^19^ Health Technology Assessors, international organizations (eg the World Health Organization) and patient representatives.^20^

The final step broadly aligns with what Black characterizes as a debate as to how the state should harness the input of non-state actors in its pursuit of public policy objectives; it is still, thus, fundamentally state-centered regulation. The four steps taken together roughly constitute the regulator-led practices of adaptation that we characterize as Adaptive Regulation.

### II.B. Adaptive Societal Governance

What, then, is Adaptive (Societal) Governance, and how does it differ from Adaptive Regulation? Firstly, we agree with the general proposition that governance is broader than regulation, and can go beyond steering the flow of events and behavior.^21^ In terms of Adaptive Governance more specifically, we support Leach et al.’s characterization that it seeks:

*‘to build capabilities based on past experiences and a commitment to social learning. Adaptive governance arrangements are conceptualized to consist of self-organizing and self-enforcing networks of individuals, organizations and agencies that have the capacity for flexible, collaborative and learning-based approaches to managing ecosystems. This means breaking away from routines that are no longer appropriate to the problem, and experimenting, adapting and reviewing new measures in a search for more resilient social– ecological relations (Folke et al., 2005). As such, adaptive governance aims to intervene in a complex socio–ecological system and guide it to some more favorable state or trajectory*’.^22^

In our present case, we would substitute the term ‘socio-ecological’ by ‘socio-technical’, but otherwise the intervention we propose is similarly aimed at creating a more resilient relationship between society and technology. This would be achieved through exploration of societal conceptualizations, to provide evidence for socially attuned updates to the laws regulating biomodifying technologies.

We agree that collaborative social learning through self-organizing networks is essential, and that this learning should take place outside the routines of regulatory practice. But the object of discussion should not be any ‘new measures’ that the regulator might adopt as part of fulfilling their functions (this could still fall under ‘Adaptive Regulation’ as steering behavior in new ways). Instead, these self-organizing networks could offer a space of reflection on the ‘foundational’ concepts that underpin regulatory frameworks, and which may be disrupted in the socio-technical interface of an emerging technology.

The box below expands upon this delineation between Adaptive Governance and our proposed concept of ‘Adaptive Societal Governance’:

**Water Basins to Eliza Doolittles: Adaptive (Societal) Governance**

The idea of ‘Adaptive’ Governance has been significantly shaped within the literature on environmental management. Characterizations vary across different papers, but a common thread is the coming together of stakeholders to manage a common resource. For example, a water basin represents a complex (but finite) socio-ecological system: it is governed by a number of laws, serves a number of purposes (eg human habitation and biodiversity) and may be threatened by uncertainty in the face of climate change. An Adaptive Governance response would see the ‘human dimensions’ of the system (laws, policies, community action) self-organize to promote the sustainable management of the basin.^23^

Our case study technologies raise an interesting question: how do you manage a ‘human’ resource, which is not finite—like a water basin—but is instead dispersed across the societal imagination? A foundational concept like ‘human nature’ is one in which all humans, by definition, have an equal ‘stake’. As such, there is no clear group of people who should be consulted as a priority when managing any challenge to, or evolution in, concepts such as ‘nature’, ‘artifice’ and ‘products’. Any disruption that threatens these conceptual resources with uncertainty, therefore, requires an approach that goes beyond ‘stakeholder’ consultation.

We therefore suggest that Adaptive ‘Societal’ Governance is the necessary next step when considering how to manage a collective resource as diffuse and nebulous as a foundational concept. While difficult to define with any degree of finality, these concepts—product, treatment, donor, device—underpin the categories we use to regulate biotechnology. They are therefore an intellectual resource vital to the governance of biomodifying technologies, even if they may appear inchoate compared to a resource from the geo-physical environment.

Consequently, a key element of ASG is that it must by necessity be multi-modal. The ambition of capturing the views of an entire society, particularly on such a qualitative question as ‘how do you understand nature, as opposed to a product’ is too broad to be realized using any single method. For example, social media studies are a promising means of capturing conceptualizations within the emerging space of the digital civil society,^24^ but it cannot be assumed that active social media users represent the general population.^25^ This approach would therefore need to be supplemented by (for example) sociological studies focusing on ‘traditional’ media that interact with other audiences, and anthropological analysis of how these concepts are understood within non-digital contexts.

Our practical suggestion as to how ASG can trace key conceptual resources across society is therefore that an interdisciplinary network of scholars could use a range of methods to observe foundational concepts as they are understood within different societies. While the primary aim of the network would not be regulatory—ie the emphasis would be on learning, rather than directing behavior—the work coming out of the network would be a useful benchmark for legislators and regulators seeking an authentic and socially responsive alignment between law and societal views.

This is not to undervalue more active forms of public engagement around the governance of emerging technologies. Measures as local as the Small Arts projects funded by the UK Wellcome Trust,^26^ or as far-reaching as the envisaged global citizens’ assembly on genome editing,^27^ are all valuable means of sharing information about biomodifying technologies, and of learning how ‘lay’ people respond to questions about (for example) the acceptable parameters of gene editing. But such work does not claim to represent society as a whole; a citizen jury of (say)25–30 people can be inclusive but not statistically representative.^28^ Jasnaoff and colleagues hope that deliberative processes can be expansive enough to avoid Eliza Doolittles having to speak like the Henry Higgenses of academia.^29^ This is indeed a valuable aim of inclusive deliberation, but a more *representative* solution would be to listen to Eliza speak in her everyday life, without inviting her to a formal engagement event in the first place.

A truly ‘foundational’ concept in Western modernity—such as that of ‘nature’ as distinct from ‘culture’—engages issues of foundational metaphysics.^30^ Resolving the question ‘what is natural?’ involves what Jasanoff terms ‘ontological surgery’—carving up ideas of the natural in an attempt to alter collective perceptions of the meaning (and boundaries) of life.^31^ It is not clear that it is the state’s role to resolve such issues, as Jasanoff asks: ‘*(i)s anyone responsible for remaking the facts of life?’*^32^ It is this inability of any one actor to monopolize the definition of ‘nature’ that motivated the establishment of Jasanoff’s Global Observatory on Genome Editing.^33^ As she and Hurlbut note, it is doubtful that existing scientific and political institutions are capable of initiating the forms of deliberation demanded by the prospect of editing life.^34^ The Global Observatory is an important step in a multi-disciplinary exchange that could form the basis for ‘social learning’ in the style of Adaptive Governance. Our suggestion, however, is that members of such networks should also report on the conceptualization of the natural/biological/modified across societies and cultures, to identify commonalities that cut across technologies, and to provide a benchmark for national regulation.

This is a preliminary, high-level account of ASG as a response to conceptual disruption. A highly detailed implementation formula would be beyond our current scope—and in any event might undermine the principle of a ‘self-organizing network’. However, a sketch of what this could look like in practical terms is attempted below:

**ASG in Action: the Digital Platform Model**

We have suggested that the Global Observatory for Genome Editing—co-led by Jasanoff, Hurlbut and Saha—covers much of the ground in which we are interested here. A fundamental commonality lies in the premise that biotechnology (in this case genome editing) raises questions about the meaning and integrity of human life which concern all of humanity, and which therefore require the greatest possible representation of perspectives across society in their attempted resolution.^35^

Our proposition differs from this existing Observatory in two main ways. Firstly, its scope is broader, as it would investigate foundational concepts underpinning the regulation of a number of technologies. For example, gene editing, bio-printing and artificial intelligence all trouble the boundary between artifice and ‘nature’, but as scholarship and policy-making tend to focus on each technology in isolation, the wider shifts in the socio-technical ecosystems—and their (dis)connection with societal understandings of ‘human nature’—may be missed. Secondly, rather than collecting insights from deliberative events, the modes of data collection would be more varied, with members of the network sharing studies from a number of disciplines, representing different populations.

As an example, an open-access digital platform could host a curated library of studies on societal understandings of (human) nature and artificial products. This would serve as the central resource around which scholars and other interested persons could share insights into conceptualizations in different societies. Some leadership would be required to create, organize and advertise the platform, but over time the submission, pursual, and dissemination of material would become the shared work of a diffuse network of users. This sharing would both consolidate existing knowledge, and also inspire further work by enabling meta-analysis of studies that would reveal the gaps in the existing literature where certain concepts—or elements of society—require further exploration.

If the above has shed light on the possible ‘how’ of ASG, the final subsection expands upon the ‘why’, with consideration of its ultimate benefit.

### II.C. The Value of ASG for Regulation

Adaptive Societal Governance is intended to operate in spaces where regulators—and even legislators—have dubious authority. No single actor can definitively determine the boundaries between nature and products when this is a question involving such broad concepts and interests. Likewise, the concept of a ‘stakeholder’ begins to fall apart when all human beings have—in a sense—an equal ‘stake’ in how we understand and manipulate ‘nature’. As the UK Nuffield Council of Bioethics has acknowledged, identifying the relevant ‘public’ to engage on gene editing is difficult when the associated rights are so universal.^36^ An ASG approach therefore involves a shift in mindset away from a ‘stakeholder’ approach and towards a ‘societal’ one, and will require study and observation of discourse outside of formal engagement spaces—such as traditional and social media as well as popular fiction.

These broader cultural phenomena may not be of immediate regulatory import; the line between a Marvel movie and EMA regulatory policy is undoubtedly indirect. But these extra-regulatory spaces are important fora for the evolution of fundamental concepts. If any link is to be established between social reflection and regulatory action, it is most likely through academic/ethnographic study of these phenomena, which could then be translated into more ‘actionable’ feedback on the changing nature of foundational concepts. This heterogeneous collection of studies would not be geared towards formulating a regulatory response to a new technology, but instead shed light onto spaces in civil society where the concepts underlying our understanding of these technologies (such as the natural or unnatural) are re-explored and reconfigured.

Even if ASG is not regulatory in its immediate intent, however, this does not preclude it from having downstream benefit for legislators and regulators. Governance of new technologies (including synthetic biological products) must go beyond considerations of its quantifiable effects,^37^ and encompass the disruption to foundational concepts. An ASG approach can inform regulatory policy and practice by using academic work to draw attention to a plurality of spaces (digital and real-world) in which shifts in foundational concepts are discussed and reflected upon. This in turn provides a broader frame of reference when trying to understand social conceptualizations of key ideas underpinning legislation.

Again, this is a high-level account of the potential value of ASG. To frame this in more concrete terms—and as a precursor to the case-studies we will explore in Section IV—the following is a brief example of a scenario in which exploration of foundational concepts could have strengthened a controversial update to UK law:

**Case Study: Mitochondrial Replacement Therapy**

An example of the potential added value of an ASG approach can be drawn from Scott and Wilkinson’s analysis of the UK and the US stance on Mitochondrial Replacement Therapy (‘MRT’).^38^ In both contexts a distinction has been drawn between gene editing and mitochondrial donation, on the basis that experts and lay people alike would consider the *nuclear* genome alone as determining ‘who we are’. Subjecting this implicit definition of human identity to scrutiny, the authors refer to Scully’s work on the narrative identity, in which she considers how a child conceived through MRT might become confused in their sense of self given their biological connection to a third person whom they may never know.^39^ In this instance, ‘identity’ is the foundational concept dividing acceptable from unacceptable biomodification. Yet the potential misalignment between social understandings of identity and its implicit framing in UK regulation undermines claims that MRT is socially and ethically acceptable because it does not have implications for ‘who we are’.

The UK’s legalization of MRT was preceded by extensive public engagement, which provided some support for the nuclear/mitochondrial distinction. Academic study later teased out some of the complexity of MRT’s social implications,^40^ but these insights came after the legislative debate. Taking an ASG approach before updating the law could have provided a more nuanced benchmark for public understandings of identity. It would involve mapping societal conceptions of human identity as a foundational concept for the regulations; for example through ethnographic observation, qualitative interviews or discourse analysis using electronic media databases.^41^ While this may or may not have changed the decision of the UK legislators to sanction MRT (which would be a *regulatory* aim to steer behavior) an understanding of ‘identity’ drawn from across society could at least have prompted a more fundamental confrontation of the ideas underlying the new law.

The three foundational concepts we consider in this paper are those of ‘naturalness’ (gene editing) a ‘product’ (bio-printing), and a ‘donor’. These are all concepts, which are deployed within legislative regulation governing advanced therapies, which is the context in which ASG is explored for this paper. Mahalatchimy and colleagues see ‘naturalness’ as a key facet of the ‘imaginary’, which frames the regulation of Advanced Therapy Medicinal Products.^42^ While we do not use the concept of the ‘imaginary’—that is, collectively held expectations about imagined futures^43^—we do draw on similar ideas of concepts that are ‘foundational’: determined socially, within and without formal regulation, and benefitting from ‘extra-regulatory’ exploration.

The next section provides an overview of the existing regulatory framework for advanced therapies and its potential for flexibility to support the translation of new therapies. This provides an illustration of Adaptive Regulation within the EU; a regulatory response that addresses the scientific and evidential uncertainty of biomodifying technologies.

## III. ADAPTIVE REGULATION

Adjustments of regulatory pathways to accommodate biomodifying technologies are not unique to the European Union, and are in evidence in many territories such as India, Japan, the United States and Australia.^44^ We focus on the EU in this paper, but the general strengths and limitations of state-orientated action (as discussed in Section II above) apply in any national context. It is therefore a detailed example of Adaptive Regulation for biomodifying technologies, which serves as a contrast for the broader remit of Adaptive (Societal) Governance.

For the most part, the EU laws, which would apply to biomodifying technologies in a medical context, are the same as for many other medical devices or medicinal products. Biomodified products may be authorized in the EU as medicinal products or medical devices, depending on their mode of function and whether they contain viable cells.^45^ This distinction has become less important within EU law since a new, third category,^46^ the ‘Advanced Therapy Medicinal Product’ (‘ATMP’), was introduced by the Advanced Therapy Medicinal Products Regulation 2007. This Regulation, known as the ATMP Regulation, is ‘lex specialis’^47^ to the Medicinal Products Directive, and seeks to bridge the gap between medical devices and medicinal products, by creating a unique category of emerging technologies and specific corresponding legal requirements. The ATMP Regulation governs a range of novel product-categories including ‘Gene Therapy Medicinal Products’, ‘Cell Therapy Medicinal Products’ and ‘Tissue Engineered Medicines’. All three of these categories of ATMPs are medicinal products, assessed by the EMA through the centralized procedure,^48^ to ensure sufficient expertise drawn from across Europe.

The EMA’s Committee on Advanced Therapies (‘CAT’) assesses ATMPs for authorization, and has representatives from each member state. Where the ATMP incorporates a medical device as a core component of its therapeutic function, it is classed as a ‘combined ATMP’, and the CAT reviews the whole product, including the assessment of the medical device by a notified body.^49^ Notified bodies are mandated by national regulators such as the UK Medicines and Healthcare Products Regulatory Agency (‘MHRA’).^50^

In the EU, medical devices are principally regulated by the Medical Device Regulation^51^ (‘MDR’), which applies from May 2021 and replaces the Medical Device Directives.^52^ The MDR establishes general obligations for manufacturers of medical devices, and sets Classification Rules^53^ that apply to medical devices, and take a risk-based approach to classification based on their potential impact on the human body. Devices will be classified as Class I, IIa, IIb, or III (low—high risk).^54^ Significantly, according to Rule 3.5, if multiple rules apply to the same device, the strictest will apply.

The ATMP Regulation does not necessarily require the strictest possible standard in its oversight of advanced therapies. It is supplemented by Good Manufacturing Practice guidelines^55^, which set out principles of quality, safety, efficacy, and traceability, similarly based on a ‘risk-based approach’.^56^ The guidelines describe standard expectations but make clear that alternative approaches can be developed if capable of meeting the same objective.^57^

These guidelines are just one example of the ways in which the applicable legislation is open to an adaptive approach by regulators—in which the precautions required are adjusted to the risk profile of the product or device in each case. The next subsection outlines some specific ways in which the ATMP Regulation and its administration are open to flexibility to accommodate emerging technologies.

### III.A. Adaptive Regulation for Biomodifying Technologies

Adaptive Regulation, as characterized in this paper, is essentially an intentionally flexible approach to a regulator’s existing functions as a response to evolving scientific knowledge.^58^ The performance of these functions can be adjusted to encompass a broader range of evidence—or perspectives—but these can only, ultimately, be tweaks to standard regulatory practice.

This is not to suggest that the role of any regulator considered in this paper has been incorrectly formulated by the relevant legislation, or that such adaptations are not helpful. The scope and focus of (for example) the EMA may well be perfectly appropriate and proportionate for their role; a role which is vitally important for safe access to medicines. Our point, rather, is that these regulatory roles are not set up to deal with the full scope of the conceptual disruption promised by new technologies. With this in mind, we consider in the next subsection the scope that the EMA is provided with, for flexibility within its legislative framework.

### III.B. Legislative Flexibility

The EU legislative framework governing medicinal products, and more specifically ATMPs, contains a number of provisions, which allow for regulatory flexibility in some circumstances. Many of these have been established by Regulation 726/2004, which sets out the ‘centralized procedure’ under which certain—more novel or complex—therapies, are approved by the EMA (including ATMPs). The options for developers seeking an element of latitude when translating a novel therapy include:^59^

**Table:** 

Provision	Legal basis	Scope	Effect
Conditional Marketing Authorization	Article 14(7), Regulation 726/2004 (the centralized procedure regulation), as inserted by Regulation 507/2006 on conditional marketing authorization.	1) Seriously debilitating or life-threatening diseases, 2) public health emergencies, 3) orphan medicinal products (see below)	A marketing authorization may be granted for one year (on a renewable basis) while further studies are conducted, with a view to standard authorization.
Exceptional Circumstances Authorization	Article 14(8), Regulation 726/2004	Where comprehensive safety & efficacy data will never be available because a) the indication is very rare **or** b) scientific knowledge is insufficient, **or** c) it would be contrary to medical ethics to collect such information.	Exceptional authorization is granted, subject to safety measures and annual review—unlikely ever to become a ‘standard’ authorization.
Orphan medicinal product designation	Initially Regulation 141/2000 on orphan medicinal products, but this has since been supplemented by a number of regulations—eg 726/2004 on the centralized procedure and regulation 1901/2006 on market exclusivity	Diseases, which are life-threatening or chronically debilitating, and which occur within no more than 5 in 10,000 people in the EU (or where it is otherwise unlikely that marketing would generate sufficient returns) *and* where there is no satisfactory method of diagnosis, prevention or treatment.^60^	Authorized products have market exclusivity for up to 12 years. Protocol assistance (ie a specific kind of scientific advice for orphan medicines) is provided to help the authorization process.
Compassionate use	Article 83 Regulation 726/2004	Life threatening, long-lasting or seriously debilitating illnesses for which there are no satisfactory authorized therapies and not all patients can enter clinical trials.	Allows the exceptional use of an unauthorized medicinal product outside clinical trials, subject to strict controls.
Accelerated assessment	Article 14(9) Regulation 726/2004	Where the product is of major interest to public health, and in particular for therapeutic innovation.	The timetable for assessment is shortened to 210 days (or 150 days if the relevant committee agrees)
Hospital exemption	Article 28 ATMP Regulation	Non-routine production of an ATMP in a hospital, under the exclusive professional responsibility of a medical practitioner.	This may be authorized by a national competent authority (instead of the European Commission via the EMA) as long as the manufacture is subject to equivalent standards.

The above table illustrates a whole raft of exceptions and adjustments available in principle to widen access for patients who would otherwise wait a long time for a new therapy. The majority are available to any medicinal product considered by the EMA through the centralized procedure—assuming the specific criteria in each case are met—but the hospital exemption and reduced cost for scientific advice is specific to ATMPs. By definition, these flexibilities only authorize products for a small population, at a small scale, or for a limited amount of time. These statutory schemes thus provide some flexibility ‘around the edges’ of the EU framework, but do not open up larger markets or establish a clear alternative to the standard marketing authorization.

### III.C. Flexible Practice: PRIME and MAPP

The EMA has explored two initiatives to try to optimize—or even expand upon—the flexibility afforded by the regulatory framework: the 2014–2016 adaptive pathways ‘MAPP’ pilot, and the PRIME priority medicines scheme. The PRIME scheme has been relatively uncontroversial, drawing as it does on the existing tools for adapting regulatory pathways outlined above (particularly Conditional Marketing Authorizations, scientific advice and accelerated assessment).^61^ As of Dec. 18, 2020, 29 of the 62 products in the scheme (47 per cent) are ATMPs, and 71 per cent are either ATMPs or biological medicinal products.^62^

The 2014–2016 MAPP pilot was more ambitious and reveals more of the issues, which can be triggered by Adaptive Regulation of new therapies. The MAPP pilot expanded upon the statutory Conditional Marketing Authorization (‘CMA’) by opening up a ‘staggered’ response to authorization to any potential products where there was an unmet clinical need, as long as a development programmer was proposed in line with the requirements to acquire real-world data throughout the process, and to discuss the evolving dataset with Health Technology Assessors.^63^ This expansion was criticized in some quarters, as the European Parliament had rejected the possibility of expanding the scope of CMA’s when the centralized procedure Regulation was debated, so in a sense the EMA was acting beyond its democratic mandate in introducing the pilot.^64^

Despite the pilot’s requirement for developers to engage with Health Technology Assessors, the perceived failure to include payers in the consultation process was identified as a key flaw in the initiative.^65^ Even in the UK, where there was evidence of broad support for these adaptive pathways, the difficulty of making reimbursement decisions on the basis of an evolving dataset was singled out as one of the greatest obstacles.^66^ Uncertainty over reimbursement—as distinct from long-term safety and efficacy—remains a significant obstacle for ATMPs.^67^ Many of these criticisms appear to have been heeded by EMA, who now state on their website:

*‘EMA is exploring the adaptive pathways concept further in the context of parallel scientific advice with HTA bodies, with the inclusion of additional stakeholders, such as patients and payer organizations’.* ^68^ (emphasis added).

This wider cast of stakeholders, operating within a formal consultation process, is a key part of the ‘inclusive’ element of Adaptive Regulation.

### III.D. National Practice

The UK provides an interesting example of an attempted adaptive approach to cell, tissue and gene therapies. A 2013 Report from the House of Lords Science and Technology Committee,^69^ called for a single source for regulatory advice for researchers, developers and manufacturers seeking to deliver regenerative therapies.

In response, a ‘One Stop Shop’ regulatory advice service for regenerative medicine was established in October 2014, which provides a single point of access to regulatory advice from the Human Fertilization and Embryology Authority (HFEA), Human Tissue Authority (HTA), MHRA, the Health Research Authority (HRA) and the UK’s health technology assessor: the National Institute for Health and Care Excellence (‘NICE’).

The One-Stop-Shop could be seen as an example of Adaptive Regulation in the UK; a more innovative approach, which nonetheless remains primarily state-orientated, regulatory, and limited to engagement with a tightly defined and product-focused set of ‘stakeholders’. To establish why adaptation needs to go beyond flexibility in the behavior of one or more regulatory agencies, the next section explores medical, social and cultural tensions caused by the interaction of our case-study technologies with EU medical law.

## IV. ADAPTIVE SOCIETAL GOVERNANCE FOR BIOMODIFICATION

As biomodifying technologies cut, merge, and transform materials at the biological level, they simultaneously cut across broader social categories, merging animal and plant, alive and dead, old and new, human and non-human. These broader categories are not restricted to scientific use, but are employed in everyday life to make sense of shared reality. As a result, biomodifying technologies and their products trouble widely held, collective meanings and boundaries, often necessitating the kinds of ‘ontological surgery’ that Jasanoff describes to try and come to terms with the new (social) realities enabled by biotechnology.^70^

We have argued in this paper that the law regulating biotechnology is premised upon ‘foundational concepts’: key shared ideas from which regulatory parameters and categories derive their meaning. We have suggested that these concepts are shared societal resources in which everyone has a stake. When biomodifying technologies disturb these concepts, ASG offers a way of mapping and tracing their construction across societies. This can in turn be used to help bring their (re)-enactment in law back in line with the evolving social imagination.

The following subsections examine how biomodification troubles established ideas of what counts as a ‘product’, what is ‘natural’ or unnatural’, and disrupts the established cultural meanings of relationships such as that between tissue ‘donor’ and recipient. The latter is considered in the next subsection.

### IV.A. iPSC: Tissues, Cells, Blood and Everything in Between

Induced pluripotent stem cells can be created by reprogramming a variety of different tissues, such as skin (fibroblasts), hair (keratinocytes), or blood (usually a type of blood cell called peripheral blood monocytes). Tissues and cells are normally collected and processed under the rules for consent, quality control, and record keeping set down by the EU ‘Tissues and Cells’ Directive^71^ while blood and blood products are subject to separate rules set out under the ‘Blood Directive’.^72^

Since the novelty of cellular reprogramming technology transcends this previously established regulatory boundary between blood and other tissue types, a developer wanting to collect starting material to make an iPSC-based cell therapy could conceivably collect material under two different sets of rules, depending on whether the cells were reprogrammed from blood or another tissue type. To address this, the UK Human Tissue Authority (HTA) and the MHRA have agreed that cell therapy developers can source material from either establishment licensed under the Blood Directive or the Tissues and Cells Directive, and that both will be equally acceptable from a regulatory point of view.^73^ This illustrates a typical, and in itself fairly successful, Adaptive Regulatory approach to the way in which iPSC technology straddles the prior regulatory boundaries between blood products, and human tissues and cells.

However, iPSC-derived blood products also occupy a ‘liminal space’^74^ between donated blood (which is often seen as natural and relatively safe by blood donors, transplant recipients, and members of the public) and synthetic blood products, which either use animal derived material or acellular chemical compounds like perfluorocarbons to transport oxygen round the body, and are often regarded as unnatural, substandard, or more risky by the public.^75^ In short, iPSC may fall in between a natural ‘donor’ relationship and an unnatural ‘synthesis’ relationship.

Disruption to foundational concepts directs attention not only to regulatory objects, but to the changing relations between people, such as tissue donors, research participants or cell therapy patients, and the world around them, through their involvement with regulated objects.^76^ IPSC-derived blood would entail a move from many blood donors worldwide to a relatively tiny number of donors contributing starting material for an industrially produced product. For some, this also blurs cultural boundaries between blood donation as an altruistic act of solidarity, where the donated blood is available to anyone who needs it, and blood as a potentially exclusive commercial product.^77^^,^^78^ Research on donation of biological materials has found that donors’ attitudes and framing of the meaning of donation varies according to what is being donated, the purpose for which donation if requested, and the identity of the recipient (whether a specific individual, a community, or an institution).^79^

This tension between understandings of donation as a transaction or as an act of altruistic solidarity is exacerbated by the potential for iPSC-derived blood, which will most likely be a high-cost product, to be targeted to subgroups defined as having high unmet need, such as patients with thalassemia or sickle cell disease (SCD). Donors of starting material for iPSC-derived blood products could be asked to show solidarity with a biological (and in the case of thalassemia and SCD, often racially or ethnically defined) population rather than to a broader communal good. ^80^ This change in the organization of donation has the potential to disrupt longstanding ideas of the donor-recipient relationship. For example, Busby^81^ has argued that, in the UK, blood donation is associated with ideas of public solidarity and a common good embodied in the National Health Service, established as part of the post-war settlement.

The case of iPSC-derived blood is thus a useful illustration of the potential for a novel technology to challenge boarder cultural meanings and values in ways that do not necessarily fall under any existing regulatory authority remit or definition of ‘risk’. This is partly because altruistic and commercial models of blood donation and the associated relationship between donor and recipients has been a particular historical object of study, making it easier to bring these questions into focus.^82^ However, the use of iPSC technology to produce other kinds of cell therapies also has the potential to trouble the meaning of relations between donor and recipient of the donated material.

One vision of the future of iPSC therapies involves biobanks of iPSC from a small number of donors selected to provide wide coverage of different human immune system markers.^83^ This would provide regulator-approved starting material, while companies would then claim the model of differentiating the cells and making and delivering a variety of end products as the commercially sensitive, IP-protected part of the process. This iPSC biobank model would again change the relationship between this limited number of, presumably uncompensated, donors, and the various ‘end users’ of the donated material.

These challenges between ‘altruistic’ models of donation and commercial models of tissue ownership are not new, as they have come up multiple times in the context of donation of material for research.^84^ However, the curative potential and high cost of iPSC cell therapies intensifies the need for debate about the equity and ethics of developing medicinal products from human bodily materials. At the core of this debate would be the role of the ‘donor’ of natural material when their bilateral relationship with the recipient is so technologically and infrastructurally mediated and disrupted.

Similarly, we previously explored the legalization of Mitochondrial Replacement Therapy in the UK, where the underlying concept of ‘identity’ was (arguably) construed narrowly in its association with the nuclear genome, and by the omission of the significance of the ‘donor’ relationship.^85^ Although we took ‘identity’ as the core concept in that scenario, we could equally have centered on the concept of a ‘donor’ as opposed to a ‘parent’—children conceived through MRT being dubbed ‘three-parent babies’ by the media.^86^ This is a case where sociological study of the scientific and ‘mass’ media could prove a helpful point of reference in mapping societal conceptions of the ‘donor vs parent’ boundary. Even if these are, in fact ‘mis’conceptions from a legal, ethical or scientific point of view, this does not mean these framings are without social consequence. How might the child in question ultimately feel if one day seeing themselves described as the product of ‘Frankinscience’ in a headline?^87^

IPSC’s are thus not the only technology putting pressure on our ideas of a ‘donor’. While they have not yet attracted the kind of media attention so far reserved for MRT, they share the common ground of having the idea of a donor-patient relationship as a core part of their process, with significant technical mediation stretching the conventional parameters of this bio-social connection. A consolidation of work examining evolving notions of a ‘donor’ in different societies (for example, through an observatory) could help us think more deeply about the long-term social re-organization both iPSC and MRT will provoke in their medical translation.

### IV.B. Bioprinting: Computer Model to Living Tissue

Bioprinting—in which layers of cells are 3D-printed through an additive process—combines software, machinery, medical devices and potential medicinal products as an end-product.^88^ It therefore offers up a complex example of regulatory convergence.^89^ At a virtual level, bioprinting begins with calculations and models applied to eg MRI scans of a body part. As bioprinting becomes more and more software-intensive, the dividing lines between these elements become blurred and final products become an incarnation of digital models.^90^ In other words, the *process* of manufacturing and the physical end *product* become increasingly indistinguishable.

Like iPSC, bioprinting challenges the division between the natural and the synthetic; in this case between the digital model and the ‘natural’, organic product. More fundamentally, however, its combination of software, cells, machine, medical device and medical expertise complicates the human-mechanical interface in a way that disrupts the very concept of a ‘product’. It is a ‘digital manufacturing’ process made up of devices and products, which are each at the cutting edge of their respective regulatory fields. Discussion of the governance of bioprinting can become dominated by somewhat repetitive debates as to whether it should be regulated as a ‘process’ or ‘product’.^91^

The debate as to whether bio-printing constitutes a medical ‘process’ or the manufacture of a ‘product’ has implications far beyond the academic sphere. Much can hinge on the legal standard of liability that flows from this characterization. In the mid-twentieth century, for example, the classification of blood transfusion as a ‘service’ rather than a ‘sale’ in the United States^92^ meant that the strict liability standard was not applied to blood as a ‘product’ in many U.S. jurisdictions, which may go some way to explaining the quality issues observed in donated blood in the 1970’s.^93^ There is therefore a policy and moral argument for understanding bio-printed material as a ‘product’ in order to afford patients the higher protection of the strict liability standard, without imputation of negligence or incompetence to the medical practitioners involved in the printing.^94^

That said, the protection afforded by product liability law should not be overestimated. In the UK—following the application of the EU Product Liability Directive—blood has been treated as a product, but the corresponding idea of a ‘defect’ is judged by what the public would be ‘entitled’ to expect—not what the public would *actually* expect—from donated blood.^95^ This means that the apparently socially responsive standard of safety in EU product liability law (‘public expectation’) can come down to a broad judicial adjudication of ‘legitimate’ expectations in ‘all circumstances’. In other words, a ‘product’ is not necessarily held to social expectations of safety (assuming product knowledge is sufficiently widespread for such expectations to exist). Hence, the law would not guarantee that a bio-printed product would conform to societal expectations of product safety, or that real-life public expectations are built into the system.

Conversely, if the classification of bio-printed human tissue *was* aligned with social conceptualizations, it should not be assumed that this would result in regulation of the material as a commercial product in the first place. Whether a bio-printed human organ would be a patentable product, and essentially capable of external ownership, is a complex question^96^ that raises questions of human dignity that EU law aims to safeguard in biotechnical regulation.^97^ Bio-printed tissue, which is structurally identical to its ‘naturally occurring’ equivalent *can* be patented if produced by means of a ‘technical process’.^98^ But the case of genetically modified organisms (‘GMO’s) illustrates the risk of creating a legally tradeable ‘product’ within the European single market when different societies across the EU have a very different cultural, religious or moral reactions to the commodification of modified life.^99^

Whether bio-printed human tissue should be treated as a product is thus a question that walks a tightrope between protecting recipient patients with the appropriate standard of liability, but also preventing the commodification of what will become a living part of their body and may even be made up of their own cells in ‘autologous’ products. This is not a simple dilemma to resolve, and an ASG investigation of biological ‘products’ as conceptualized in different societies would be a welcome resource in aligning a solution with broader social ideas of nature and production.

In our final example, it is more directly the idea of the ‘natural’ itself, which is the disturbed foundational concept at stake. We will consider this in the next sub-section.

### IV.C. Gene Editing: Recombination vs the ‘Natural’

Like iPSC and bio-printed products, gene-editing agents occupy a liminal space between the natural and the unnatural. The relevant space is not just cultural but regulatory, created by the subsisting distinction in the EU between the ‘natural’ and the ‘modified’, which does not take into account ‘natural’ products, which have been made synthetically. CRISPR—derived as it is from a naturally occurring feature in bacterial immune systems—is not necessarily captured by the ‘recombination’ criterion in the ATMP Regulation, which stems from a societal controversy over GMO’s, which may no longer be the pressing consideration for this technology.

As Martin and colleagues have highlighted,^100^ behind the apparently explosive proliferation of CRISPR in recent years are a number of supporting technologies and infrastructures. The speed of genetic sequencing has dramatically reduced over the last twenty, even ten, years, making targeted gene editing a far more realistic prospect. The development of machine-learning has been claimed to improve the accuracy with which off-target effects of gene editing can be calculated,^101^ bolstering the claim for the safety of the intervention. Resources such as Addgene, which existed before CRISPR became widely available, are now key factors in its commercial accessibility.^102^

Libraries of industrially manufactured CRISPR, and other types of gene-editing reagents, are clearly key drivers in the development of this technology. Industrial synthesis of otherwise natural molecules^103^ highlights a significant lacuna in EU regulation of gene therapies. As noted, the ATMP Regulation covers gene therapy medicinal products (‘GTMP’),^104^ defined as a biological medical product (ie a medicinal product whose active substance is produced by or extracted from a biological source^105^) that

(a) contains an active substance, which contains or consists of a recombinant nucleic acid used in or administered to human beings with a view to regulating, repairing, replacing, adding or deleting a genetic sequence;
(b) its therapeutic, prophylactic or diagnostic effect relates directly to the recombinant nucleic acid sequence it contains, or to the product of genetic expression of this sequence.

As two of the authors have argued elsewhere,^106^ the ‘recombination’ element of the definition risks excluding many gene-editing molecules, which have been produced synthetically. Recombination is not defined in the ATMP Regulation, but from the Directive governing GMO’s (‘GMO Directive’) we can infer that the definition is:

‘*techniques involving the formation of new combinations of genetic material by the insertion of nucleic acid molecules produced by whatever means outside an organism into any virus, bacterial plasmid or other vector system and their incorporation into a host organism in which they do not naturally occur but in which they are capable of continued propagation’*.^107^

In other words, when the ‘naturally occurring’ DNA has an additional section of nucleic acid added to it to create a new DNA sequence that does not occur in nature, the result is genetic modification. This idea of modifying the natural into the unnatural is central to the GMO Directive, but is out of step with a world in which the ‘natural’ can be produced by artificial processes.

The emphasis on recombination makes sense in the GMO Directive, drafted as it was at a time when the Cartagena Protocol (which the Directive implements^108^) defined ‘modern biotechnology’ as including rDNA techniques.^109^ It is evident, however, that biotechnology has progressed in the last 20 years: where a zinc-finger nuclease (for example) is delivered without recourse to a recombinant viral vector, gene editing may be achieved without using nucleic acid molecules at all. Even if CRISPR (or its variants^110^) were used, it may still be generated synthetically and without adding and recombining new genetic material in the editing molecule. In this case, the molecule would be out of scope the ATMP Regulation.

The natural vs modified binary—as constituted in the ATMP Regulation—no longer reflects technological possibilities, in particular the potential for ‘natural’ molecules to be produced synthetically, or to interfere with the nature of the human body. This is a distinct part of a broader trend in which the natural/unnatural line is challenged by biological medicine. A parallel can be seen in the emergence of epigenetics alongside genomic medicine. The discovery of DNA in the 1950’s ushered in what Jasanoff terms ‘the genetic age’, in which law and biology have interacted in a mutually constitutive way to define the moral and physical ideas of what natural life is.^111^ Even this, however, is potentially surpassed by new understandings of epigenetics—of nature as dynamic and environmentally constituted, as shifts in the expression of DNA prove increasingly more significant relative to the static content of the DNA itself.^112^ Life itself—in other words—is a process of gene editing.

It is, perhaps, no longer accurate to conceive of an innate, stable ‘nature’ in biological phenomena, in which a modification is an easily identifiable ‘artifice’. The arguably anachronistic exceptionalism accorded to DNA molecules, and thus to corresponding ‘genetic data’, has led to gaps in the law whereby ‘epigenetic discrimination’ is not yet recognized as a risk, as the significance of these environmental modifications is not widely understood.^113^ In many ways, the law continues to protect a stable version of biological nature that may not exist in reality. However, as with so-called ‘three parent babies’ (see Section IV.A), even an arguable ‘misconception is still important if it holds sway with a significant proportion of society. Even if we can argue that the evolution of scientific knowledge has stripped the nature-artifice boundary of reliable meaning, this does not mean the law should automatically be updated to reflect expert insight. If—as the case of GMO’s suggests—many people in society still have a strong attachment to the idea of “nature” as a default state of purity, which requires preservation, this remains relevant for an ASG account of this foundational concept. This conceptualization of ‘nature’ would remain an important part of the social imagination through which the acceptability of the law will inevitably be judged.

A diffuse network operating in an observatory model could potentially be well-placed to investigate and exchange insight into societal constructions of a concept as broad as ‘nature’. As Jasanoff argues:

‘*(r)evolutions in our understanding of what life is burrow so deep into the foundations of our social and political structures that they necessitate, in effect, a rethinking of law at a constitutional level’.*

We concur with the profundity of the disruption depicted, although we are concerned here with underlying foundational concepts rather than legal constitutions. While legislative texts such as the ATMP Regulation play a ‘performative’ role in shaping conceptions of technology,^114^ the ontological, societal implications are oblique, concealed behind legal language and certainly not readily contestable. We therefore endorse empirical work bridging the social and the regulatory in mapping out disruption and evolution in the idea of the ‘natural’ and other foundational concepts. To represent the greatest possible sweep of the social imagination, a self-organizing network exchanging learnings on this concept will need to be open to society through inclusive study, as well as inclusive deliberation.

## V. CONCLUSION

Biomodifying technologies threaten to disturb the key concepts on which their relevant regulatory frameworks are based. We have proposed Adaptive ‘Societal’ Governance as a response to this disruption. ‘ASG’ is an adjustment to the ‘Adaptive Governance’ approach conceived of within environmental management, as the latter was conceived in response to analogous challenges. As with a ‘natural’ resource, foundational concepts like ‘product’ and ‘nature’ are widely shared, serve multiple functions, and cut across regulatory systems in multiple national contexts. Like climate change in the physical environment, technology-driven disruption to our collective conceptual understandings of the world can render what was seen as stable and immutable, uncertain and fraught.

In response to this conceptual uncertainty, ASG would promote social learning through a self-organizing network. However, rather than bringing people together in a formal ‘engagement’ exercise, or ‘deliberative’ discussion, the ‘learning’ of this network should be drawn from outside itself, with multiple empirical methods used to trace foundational concepts across societies.

A global observatory-style network with (for example) a digital platform as a central point of organization could bring together studies of foundational concepts, which already take place in sociology, anthropology and across the humanities. These studies should cut across technologies, and specific regulatory questions. Gene editing, bio-printing and even artificial intelligence all challenge our idea of ‘nature’ as opposed to ‘artifice’; a broader scope could reveal the common threads in associated social thinking which siloed focus on specific technology might miss.

We recommend that this network should revolve around ongoing sharing (potentially through said open-access digital platform) of studies of foundational concepts in different societies. In doing so, we could not only consolidate and disseminate existing knowledge, but also identify gaps in the literature; perhaps where the views of certain sections of society have been under-represented and require more exploration.

This approach could be valuable in its downstream impact, but also in its own right. Of course, by providing better benchmarks for societal conceptualizations, ASG could be a resource for legislators, regulators and anyone else taking responsibility for ‘remaking the facts of life’.^115^ It could even bolster any update to the relevant legislation with the depth, authenticity and legitimacy of alignment with extra-regulatory constructions of the concepts around which such laws are formulated, thus making them more resilient to the disruption biomodifying technologies will bring.

However, we also agree with the premise of Jasanoff’s Global Observatory—that widespread reflection on the (re)definition of biological life and nature is a whole-of-humanity issue.^116^ Insight into how these concepts are (re)imagined can therefore be seen as important in its own right. While we might want to see ASG learnings form part of the regulatory fabric around emerging technologies, the line between the ‘bio’ and the ‘modified’ is a question on which all biological persons have an equal ‘stake’. An open-access platform is therefore one way of ensuring that an ASG network is open to all interested persons in the societies from which it draws its findings, and its benefits are shared as widely as the multiple fora and discourses from which it seeks to learn.

## Footnotes

1
Hannah Landecker, Culturing Life: How Cells Became Technologies (2010).

2 Rosamund Scott and Stephen Wilkinson, *Germline Genetic Modification and Identity: the Mitochondrial and Nuclear Genomes,* 37 Oxf. J. Leg. Stud. 886 (2017).

3 See Scott and Wilkinson (*Id.*) on the framing of ‘identity’ in UK law governing mitochondrial donation vs social understandings of identity; discussed in Section II.

4 Sheila Jasanoff and J. Benjamin Hurlbut, *A Global Observatory for Gene Editing,* 555 Nature 435 (2018).

5
*Id.* at 437.

6 Simon Burall, *Don’t Wait for An Ooutcry About Gene Editing,* 555 Nature 438 (2018).

7 The term ‘Planned Adaptive Regulation’ has been explored in more depth by the International Risk Governance Council—see for example IRGC, *A Short Introduction to ‘Planned Adaptive Regulation’*, https://irgc.org/wp-content/uploads/2018/09/A_short_introdution_to_Planned_Adaptive_Risk_Regulation-19Nov15.pdf (accessed Jan. 26, 2022). We use the term in a similar way, to encompass *ex ante,* intentional flexibility on the part of regulators in response to evolving scientific knowledge.

8 Oliver Feeney et al., *Patenting Foundational Technologies: Lessons From CRISPR and Other Core Biotechnologies*, 18 Am. J. Bioeth. 36 (2018); Michael Morrison and Stevie De Saille, CRISPR in *Context: Towards a Socially Responsible Debate on Embryo Editing*, 5 Palgrave Commun 110 (2019).

9
Landecker, *supra* note 1. See also Michael Morrison et al., *Biomodifying Technologies and Experimental Space: Organisational and Regulatory Implications for the Translation and Valuation of Health Research*, 1 Impact 63 (2019).

10 A range of different gene-editing tools including ‘zinc finger nucleases’ (ZFN), transcription activator-like effector nucleases (TALENs) and CRISPR/cas9 (where CRISPR stands for ‘clustered randomly interspaced short palindromic repeats’) are currently in use, and it is not unreasonable to assume more techniques will be developed over time. Some noteworthy recent techniques include base editing and PRIME editing. Base editing uses a CRISPR system with a modified ‘cas9’ enzyme to change single ‘bases’- the letters of the DNA code (A, G, C and T) instead of cutting the DNA. PRIME editing is a further modification of CRISPR that uses a different modified cas9 enzyme to insert new DNA sections without making a double stranded break in the DNA. It is viewed as more efficient and less risky because it does not require a double stranded break in the DNA to insert new material but uses a ‘nick’ in a single strand to add new genetic material. See eg Janine Scholefield and Patrick T. Harrison, *Prime editing—an update on the field,* 28 Gene Therapy 396 (2021).

11 Paul Martin et al., *Genome Editing: The Dynamics of Continuity, Convergence, and Change in the Engineering of Life*, 39 New Genet. Soc. 219 (2020).

12 Neil Stephens et al.*, Bringing Cultured Meat to Market: Technical, Socio-political, and Regulatory Challenges in Cellular Agriculture*, 78 Trends Food Sci. Technol. 155.

13 European Commission, Final Report Summary, *EUCELLEX (Cell-based Regenerative Medicine: New Challenges for EU Legislation and Governance)*, https://cordis.europa.eu/project/id/601806/reporting (accessed Jan. 26, 2022).

14 Julia Black, *Critical Reflections on Regulation* 27 Aust. J. Legal Philos. 1 (2002).

15 Dimity Kingsford Smith, *What Is Regulation—A Reply to Julia Black*, 27 Aust. J. Legal Philos. 37 (2002).

16
Mark Bevir, A Theory of Governance, 1 (2013).

17 Most crucially, for our present purposes, the European Medicines Agency, which includes representation from a number of EU member states.

18 For example, notified bodies play a key role in medical device regulation even though they are part of the private sector, and often multi-national in scope.

19 Patricia Vella Bonanno et al., *Adaptive Pathways: Possible Next Steps for Payers in Preparation for Their Potential Implementation*, 8 Front. Pharmacol. 497 (2017).

20 These were the categories of stakeholder identified by the EMA in their 2016 report, and the associated commentary also focuses on these as the main categories. See European Medicines Agency, *Final Report on the Adaptive Pathways Pilot,* 28 July 2016, available from https://www.ema.europa.eu/en/documents/report/final-report-adaptive-pathways-pilot_en.pdf, (accessed Jan. 26, 2022).

21 John Braithwaite, Cary Coglianese, and David Levi-Faur, *Can Regulation and Governance Make a Difference?*, 1 Regul. Gov. 1 (2017).

22
Melissa Leach, Ian Scoones, Andy Stirling, Dynamic Sustainabilities: Technology, Environment, Social Justice (2010).

23 Barbara Cosens et al., *The Adaptive Water Governance Project: Assessing Law, Resilience and Governance in Regional Socio-Ecological Water Systems Facing a Changing Climate,* 51 Idaho L. Rev. 1 (2014).

24 Matthew L Williams et al., *Towards an Ethical Framework for Publishing Twitter Data in Social Research: Taking into Account Users’ Views, Online Context and Algorithmic Estimation* 51 Sociology 6 (2017).

25 Luke Sloan, *Who Tweets in the United Kingdom? Profiling the Twitter Population Using the British Social Attitudes Survey 2015,* 3 Soc. Media Soc. (2017).

26 Wellcome Trust, *Grants Awarded: Small Arts Awards*https://wellcome.org/grant-funding/funded-people-and-projects/small-arts-awards-people-funded (accessed Jan. 27, 2022).

27 John S. Dryzek et al., *Global Citizen Deliberation on Genome Editing,* 369 Science 1435 (2020).

28 Donrich Thaldar et al., *A Virtual Deliberative Public,Engagement Study on Heritable Genome Editing among South Africans: Study Protocol*, 16 PLoS One 8 (2021).

29 J. Benjamin Hurlbut et al., *CRISPR Democracy: Gene Editing and the Need for Inclusive Deliberation*, 32 Issues Sci. Technol. 25 (2015).

30 Yrjö Haila, *Beyond the Nature-Culture Dualism*, 15 Biol. Philos. 155 (2000).

31 Sheila Jasanoff, *Making the Facts of Life,* in Reframing Rights: Bioconstitutionalism in the Genetic Age 59 (Sheila Jasanoff ed., 2011).

32
*Id.* at 60.

33 Global Observatory, *About,*https://global-observatory.org/about/ (accessed Jan 26, 2022).

34 Jasanoff and Hurlbut, *supra* note 4.

35 Global Observatory, *supra* note 33.

36 Nuffield Council on Bioethics, *Genome Editing: An Ethical Review* (2016), as cited in Scott and Wilkinson, *supra* note 2, at 894.

37 Igor Linkov et al., *Comparative, Collaborative, and Integrative Risk Governance for Emerging Technologies*, 38 Environ. Syst. Decis. 170 (2018).

38 Scott and Wilkinson, *supra* note 2.

39 Jackie Leach Scully, *A Mitochondrial Story: Mitochondrial Replacement, Identity and Narrative*, 31 Bioethics 37 (2017), as cited in Scott and Wilkinson (*supra* note 2).

40 Ilke Turkmendag, *It Is Just a ‘Battery’: ‘Right’ to Know in Mitochondrial Replacement,* 43 ST&HV 56 (2017); Cathy Herbrand and Rebecca Dimond, *Mitochondrial Donation, Patient Engagement and Narratives of Hope* 40 Sociol Health Illn 623 (2017).

41 For an example of discourse analysis as a means of investigating social concepts in healthcare, see Shan Mohammed et al., *The ‘Nurse as Hero’ Discourse in the COVID-19 Pandemic: A Poststructural Discourse Analysis*, 117 Int J Nurs Stud 103887 (2021).

42 Aurelie Mahalatchimy, Pin Lean Lau, Phoebe Li, and Mark Flear, *Framing and Legitimating EU Legal Regulation of Human Gene-Editing Technologies: Key Facets and Functions of an Imaginary,*J. Law Biosci. (2021) (forthcoming).

43
Jens Beckert, Imagined Futures: Fictional Expectations and Capitalist Dynamics (2016).

44 Shashank S Tiwari et al., *Regenerative Medicine in India: Trends and Challenges in Innovation and Regulation*, 12 Regen Med 875 (2017); Yoji Jokura et al., *Comparison of the new Japanese legislation for Expedited Approval of Regenerative Medicine Products with the Existing Systems in the USA and European Union,* 12 J Tissue Eng Regen Med e1056 (2017); Jane Nielsen et al., *Bespoke Regulation for Bespoke Medicine? A Comparative Analysis of Bioprinting Regulation in Europe, the USA and Australia,* 5 3D Print Med 155 (2021).

45 Cells are not viable under the Medical Devices Regulation (Article 2(16)) if they have no potential for metabolism or multiplication.

46 Tania Bubela et al., *Bringing Regenerative Medicines to the Clinic: the Future for Regulation and Reimbursement*, 10 Regen. Med. 897, 902. (2015).

47 Phoebe Li, Alex Faulkner, and Nicholas Medcalf *3D Bioprinting in a 2D Regulatory Landscape: Gaps, Uncertainties and Problems* 12 Law Innov. Technol. 1 (2020).

48 As governed by Regulation 726/2004.

49 ATMP Regulation, Article 9(3).

50 The MHRA no longer operates under the wider EU framework, but as it has done so for most of its existence, it can still illustrate some of the broader points explored in this paper.

51 Regulation (EU) 2017/745 of the European Parliament and of the Council of Apr. 5, 2017 on medical devices, amending Directive 2001/83/EC, Regulation (EC) No 178/2002 and Regulation (EC) No 1223/2009 and repealing Council Directives 90/385/EEC and 93/42/EEC (Text with EEA relevance.).

52 Council Directive 93/42/EEC of June 14, 1993 concerning medical devices.

53 MDR, Annex VIII sets out the risk classification criteria.

54
*Id.*

55 European Commission, *Guidelines on Good Manufacturing Practice specific to Advanced*
*Therapy Medicinal Products*, https://ec.europa.eu/health/sites/health/files/files/eudralex/vol-4/2017_11_22_guidelines_gmp_for_atmps.pdf (accessed Jan. 26, 2022)

56
*Id*. at.8.

57
*Id*. at 6.

58 See the International Risk Governance Council, *supra* note 7.

59 Lynn G. Baird et al., *Accelerated Access to Innovative Medicines for Patients in Need*, 96 Clin. Pharmacol. Ther. 559. (2014); Gayathri Balasubramanian et al., *An Overview of Compassionate Use Programs in the European Union Member States,* 5 Intractable Rare Dis. Res. 244 (2016).

60 If such a treatment does exist, the new therapy must be of significant benefit to patients.

61 Giulia Detela and Anthony Lodge, *EU Regulatory Pathways for ATMPs: Standard, Accelerated and Adaptive Pathways to Marketing Authorisation*, 13 Mol. Ther. Methods Clin. Dev. 205 (2019).

62 European Medicines Agency, *PRIME: Priority Medicines*, https://www.ema.europa.eu/en/human-regulatory/research-development/prime-priority-medicines (accessed Jan. 26, 2022)

63 European Medicines Agency, *Final Report on the Adaptive Pathways Pilot*, https://www.ema.europa.eu/en/documents/report/final-report-adaptive-pathways-pilot_en.pdf (accessed Jan. 26, 2022).

64 Keith Syrett, *Regulation, Innovation and Disruption: the European Medicines Agency and Adaptive Licensing of Pharmaceuticals*, 12 Law Innov. Technol. 2 (2020).

65 Patricia Vella Bonanno et al., *Adaptive Pathways: Possible Next Steps for Payers in Preparation for Their Potential Implementation*, 8 Front. Pharmacol. 497 (2017).

66 Office for Life Sciences, *Medicine Adaptive Pathways for Patients: A Report on Ethical Issues, Real World Data and the Views of Patients and Professionals by the Centre for the Advancement of Sustainable Medical Innovation (CASMI)*https://assets.publishing.service.gov.uk/government/uploads/system/uploads/attachment_data/file/509647/mapps.pdf (accessed Jan. 27, 2022).

67 Bubela et al., *supra* note 46.

68 European Medicines Agency, *Adaptive Pathways*https://www.ema.europa.eu/en/human-regulatory/research-development/adaptive-pathways (accessed Jan. 26, 2022).

69 House of Lords, *Regenerative Medicine Report,*https://publications.parliament.uk/pa/ld201314/ldselect/ldsctech/23/23.pdf (accessed Jan. 26, 2022).

70 Jasanoff, *supra* note 31.

71 Directive 2004/23/EC of the European Parliament and of the Council of 31 March 2014 on setting standards of quality and safety for the donation, procurement, testing, processing, preservation, storage and distribution of human tissues and cells.

72 Directive 2002/98/EC of the European Parliament and of the Council of Jan. 27, 2003 setting standards of quality and safety for the collection, testing, processing, storage and distribution of human blood and blood components and amending Directive 2001/83/EC.

73 Catapult Cell and Gene Therapy, *HTA Provides Statement on Regulation of Blood as a Starting Material for ATMPs*, https://ct.catapult.org.uk/news-media/regulatory-news/hta-provides-statement-regulation-blood-starting-material-atmps (accessed Jan. 26, 2022).

74 Graeme Laurie, *Liminality and the Limits of Law in Health Research Regulation: What are we Missing in the Spaces in-Between?* 25 Med. Law Rev. 47 (2017); Nayha Sethi, *Regulating for Uncertainty: Bridging Blurred Boundaries in Medical Innovation, Research and Treatment*, 11 Law Innov. Technol. 112 (2019); Samuel Taylor-Alexander, *Beyond Regulatory Compression: Confronting the Liminal Spaces of Health Research Regulation,* 8 Law Innov. Technol. 149 (2016).

75 Emma King and Catherine Lyall, *What’s in a Name: Are Cultured Red Blood Cells ‘Natural’?,* 40 Sociol. Health Illn. 687 (2018).

76 Taylor-Alexander et al., *supra* note 74.

77 Emma King, *Biodiversity and Perceptions of Risk: Reactions to the Use of a Single Donor for Stem-Cell-Derived Red Blood Cell Transfusions*, 3 J Law Med. Ethics 151 (2015).

78 Julie Kent and Darian Meacham, *Synthetic Blood’: Entangling Politics and Biology*, 25 Body Soc. 28 (2019).

79 Gulam Bahadur, Michael Morrison, and Laura Machin, *Beyond the ‘Embryo Question’: Human Embryonic Stem Cell Ethics in the Context of Biomaterial Donation in the UK*, 21 Reprod. Biomed. Online 868 (2010).

80 Kent and Mecham, *supra* note 78.

81 Helen Busby, *Biobanks, Bioethics and Concepts of Donated Blood in the UK*, Sociol. Health Illn. 850 (2006).

82
Richard Titmuss, the Gift Relationship: From Human Blood to Social Policy. (1970).

83 Ian Wilmut et al., *Development of a Global Network of Induced Pluripotent Stem Cell Haplobanks*,10 Regen. Med. 235 (2015); Jacqueline Barry et al., *Setting Up a Haplobank: Issues and Solutions,* 1 Curr. Stem Cell Rep. 110 (2015).

84 Gerald Dworkin and Ian Kennedy, *Human Tissue: Rights in the Body and Its Parts*, Med. Law Rev. 291; Lori Andrews & Dorothy Nelkin, Body Bazaar: The Market for Human Tissue in the Biotechnology Age (2001); Donna Dickenson, Body Shopping: The Economy Fuelled by Flesh and Blood (2008); Catherine Waldby and Robert Mitchell, Tissue Economies: Blood, Organs and Cell Lines in Late Capitalism (2006); Gulam Bahadur and Michael Morrison, *Patenting Human Pluripotent Cells: Balancing Commercial, Academic and Ethical Interests*, 25 Hum. Reprod. 14 (2010).

85 See Section II.

86 See, for example, Jo Macfarlane, *Has Britain’s First Three-Parent Baby Already Been Born?,*https://www.dailymail.co.uk/health/article-10274893/Has-Britains-three-parent-baby-born.html (accessed Jan. 27. 2022).

87
*Id.*

88 Samad Ahadian and Ali Khademhosseini, *A Perspective on 3D Bioprinting in Tissue Regeneration*, 1 Bio-Des. Manuf. 157 (2018); Guifang Gao et al., *Organ Bioprinting: Are We There Yet?*, 7 Adv. Healthc Mater. 1. (2018); Christian Mandrycky et al., *3D Bioprinting for Engineering Complex Tissues*, 34 Biotechnol. 422 (2016).

89 Phoebe Li, Alex Faulkner, Nicholas Medcalf, *supra* note 47.

90 For example, at Newcastle University, a group of researchers produced the world’s first bioprinted human cornea. The resulting cornea would not be functional without having exact shape and configuration. The proprietary AutoCAD software package was used to model not only the cornea but also a support structure that maintains the tissue’s shape during the printing process. To link the computer model and the bioprinter, the open-source Slic3r software was used. Abigail Isaacson, Stephen Swioklo, Stephen and Che J. Connon, *3D Bioprinting of a Corneal Stroma Equivalent*, 173 Exp. Eye Res. 173 (2018).

91 Phoebe Li, Alex Faulkner, Nicholas Medcalf, *supra* note 47.

92 By the New York Court of Appeals in *Perlmutter v Beth David Hosp*., 123 N.E.2d 792 (N.Y. 1954), cited by Havinghurst (below).

93 Clark C. Havighurst, *Trafficking in Human Blood: Titmuss (1970) and Products Liability*, 72 Law & Contemp. Probs. 1 (2009).

94
*Id.* at 3.

95
*A v National Blood Authority* [2001] 3 All ER 289 at [31].

96 Jasper L. Tran, *Patenting Bioprinting,* JOLT (2015).

97
Directive 98/44/Ec of the European Parliament and of the Council of 6 July 1998 on the legal protection of biotechnological inventions.

98
*Id.,* Article 3.

99 Maria Lee, *GMOs in the Internal Market: New Legislation on National Flexibility*, 79 Mod. L. Rev. 317 (2016).

100 Martin et al., *supra* note 11.

101 Qiaoyue Liu et al., *Deep Learning Improves the Ability of sgRNA Off-Target Propensity Prediction,* 21 BMC Bionformatics 51 (2020).

102 Martin et al., *supra* note 11.

103 Martin Jinek et al., *A Programmable Dual-RNA-Guided DNA Endonuclease in Adaptive Bacterial Immunity*, 337 Science 816 (2012).

104 ATMP Regulation Article 2(1)(a), as defined in Part IV of Annex I to Directive 2001/83/EC.

105 Medicinal Product Directive, para 3.2.2.1.

106 Miranda Mourby and Michael Morrison, *Gene Therapy Regulation: Could In-Body Editing Fall Through the Net?*, 28 Eur. J. Hum. Genet. 979 (2020).

107 Council Directive 2001/18/EC of 12 March 2001 on the deliberate release into the environment of genetically modified organisms and repealing. Council Directive 90/220/EEC [2001] OJ L 106/1, Annex 1A, Part 1, para (1).

108
*Id*, Article 32.

109 Secretariat of the Convention on Biological Diversity (2000). Cartagena Protocol on Biosafety to the Convention on Biological Diversity, Article 3(2).

110 See *supra* note 10.

111 Jasanoff, *supra* note 31.

112 Maurizio Meloni and Giuseppe Testa, *Scrutinizing the Epigenetics Revolution*, 9 Biosoc. 431 (2014).

113 Stephanie Dyke et al., *Points-to-Consider on the Return of Results in Epigenetic Research*, 11 Genome Med. 31 (2019); Charles Dupras et al., *Epigenetic Discrimination: Emerging Applications of Epigenetics Pointing to the Limitations of Policies Against Genetic Discrimination*, 9 Front. Genet. 202 (2018).

114 Alex Faulkner, *Law’s Performativities: Shaping the Emergence of Regenerative Medicine through European Union Legislation*, 42 Soc. Stud. Sci. 753 (2012).

115 Jasanoff, *supra* note 31.

116 Global Observatory, *supra* note 33.

